# Genetic improvement of speed across distance categories in thoroughbred racehorses in Great Britain

**DOI:** 10.1038/s41437-023-00623-8

**Published:** 2023-05-27

**Authors:** Patrick Sharman, Alastair J. Wilson

**Affiliations:** Centre for Ecology and Conservation, University of Exeter (Penryn Campus), Cornwall, TR10 9FE UK

**Keywords:** Evolutionary genetics, Animal breeding, Quantitative trait

## Abstract

Several studies over recent decades have reported a lack of contemporary improvement in thoroughbred racehorse speed, despite apparent additive genetic variance and putatively strong selection. More recently, it has been shown that some phenotypic improvement is ongoing, but rates are low in general and particularly so over longer distances. Here we used pedigree-based analysis of 692,534 records from 76,960 animals to determine whether these phenotypic trends are underpinned by genetic selection responses, and to evaluate the potential for more rapid improvement. We show that thoroughbred speed in Great Britain is only weakly heritable across sprint (*h*^2^ = 0.124), middle-distance (*h*^2^ = 0.122) and long-distance races (*h*^2^ = 0.074), but that mean predicted breeding values are nonetheless increasing across cohorts born between 1995 and 2012 (and racing from 1997 to 2014). For all three race distance categories, estimated rates of genetic improvement are statistically significant and also greater than can be explained by drift. Taken together our results show genetic improvement for thoroughbred speed is ongoing but slow, likely due to a combination of long generation times and low heritabilities. Additionally, estimates of realised selection intensities raises the possibility that the contemporary selection emerging from the collective actions of horse breeders is weaker than previously assumed, particularly over long distances. We suggest that unmodelled common environment effects may have upwardly biased estimates of heritability, and thus expected selection response, previously.

## Introduction

Thoroughbreds are the most widely used breed of horse in racing globally. Given the financial incentives and the highly competitive nature of the thoroughbred racehorse breeding industry, it is no surprise that estimates of selection on these horses (based on Timeform handicap ratings, a widely used measure of racehorse performance in Great Britain and Ireland) have been high for stallions (top 2–6% selected), though less for dams (top 44–61% selected) given they can only produce one offspring each year (More O’Ferrall and Cunningham 1974; Field and Cunningham 1976; Gaffney and Cunningham 1988).

Despite putatively strong selection and the perceived importance of genetics, a number of studies have failed to detect substantive improvement in thoroughbred racehorse speed over recent decades (Cunningham 1975; Gardner 2006; Denny 2008; Desgorces et al. 2012). This in turn has led to the suggestion that thoroughbred performance has reached a *de facto* selection limit (discussed in Hill 1988; Eckhardt et al. 1988; Simm et al. 2004; Denny 2008; Desgorces et al. 2012), perhaps due to anatomical or physiological constraints (Simm et al. 2004; Gardner 2006), associated with increased incidence of physical disorders (Holden 1991) or susceptibility to injury (Drape 2008; Mitchell 2008; Gibbons 2014). In fact, in a more recent and much more comprehensive analysis of race times recorded from the mid-1800s to 2012, we showed that racehorses are still getting faster in races in Great Britain (i.e., England, Wales and Scotland; henceforth GB; Sharman and Wilson 2015). However, the recent improvement detected was largely driven by increases in running speed over short-distance races. Notably, rates of phenotypic improvement across all distances are well below those routinely achieved in livestock breeding programmes (Hill 2016).

For a single target trait under simple mass selection, the expected rate of genetic improvement in the trait mean is equal to the product of the strength of selection (as measured by the selection differential) and the trait heritability (h^2^_,_ the proportion of phenotypic variance attributable to additive genetic variance; Falconer and Mackay 1996). Unfortunately, uncertainty persists over both key parameters for speed in racehorses. Firstly, while it is difficult to argue that faster horses are not preferred on average, speed itself is certainly not the only target of selection (Langlois 1996). Secondly, though many studies have estimated genetic variance for performance in thoroughbreds (More O’Ferrall and Cunningham 1974; Langlois 1980; Hintz 1980; Gaffney and Cunningham 1988; Oki et al. 1995; Williamson and Beilharz 1998; da Mota et al. 2005; Ekiz and Kocak 2007; Bakhtiari and Kashan 2009; Tozaki et al. 2012; Velie et al. 2015a; Velie et al. 2015b) and other racehorse breeds (e.g., Willham and Wilson 1991; Ekiz et al. 2005; Corrêa and da Mota 2007; Faria et al. 2019), published heritability estimates vary considerably. While heritability is of course a population specific parameter, part of this variation likely reflects imprecision arising from the low sample sizes used by some studies. In addition there are also differences in statistical methodology applied, the categories of race included (e.g., with respect to distance, horse age), and the structure of the data analysed (e.g., winning times only vs finishing times of all runners in a race). Additionally, many different measures of performance have been used (e.g., race time or speed, finishing position, handicap rating, prize earnings etc.). However, even restricting to studies where time (or speed) of all finishers was used to assay thoroughbred performance, reported heritabilities range substantially (Oki et al. 1995; da Mota et al. 2005; Ekiz and Kocak 2007; Bakhtiari and Kashan 2009; Velie et al. 2015b).

As a consequence of the above areas of uncertainty, it is difficult to assess at present whether the comparatively low rates of contemporary phenotypic improvement in racehorse speed are a consequence of inefficient selection, a lack of additive genetic variance, or both. Importantly, the ongoing phenotypic improvement need not reflect any genetic improvement at all since changes in average race speed are also known to arise from non-genetic factors. These include known changes in jockey riding posture (Pfau et al. 2009) and race timing methods (Sharman and Wilson 2015) in the past. It is also intuitive that continual development of training methods, animal nutrition and veterinary care will positively impact performance too. Thus, the goal of the current study is to determine whether, and to what degree, contemporary improvement in thoroughbred racehorse finishing times reflects a genetic response to selection for increased speed in Great Britain.

## Materials and methods

### Race records and pedigree structure

Performance records were supplied by TBGenerations Limited and we included only ‘flat’ races (i.e., not those with jumps) run on ‘turf’ (i.e., not those run on artificial surfaces) on GB racecourses. The full dataset comprises 692,534 race times in races held from 1997 to 2014. Given our previous finding that rates of phenotypic improvement in racehorse speed differed across categories of race distance (Sharman and Wilson 2015), we defined three subsets of this data that contain records from races in defined distance windows. These were: *sprint* races of 5–7 furlongs; *middle-distance* races of 8–12 furlongs; and *long-distance* races of 14–20 furlongs. Note that we use non-SI units as these are standard in thoroughbred horseracing; there are 220 yards in a furlong and 1 yard = 0.9144 m. A pedigree structure associated with the full dataset was supplied by TBGenerations Limited. The full pedigree contains 106,447 horses. To reduce computing time, when analysing the different race distance data subsets the full pedigree was ‘trimmed’ to remove individuals not informative for genetic parameter estimation. Informative individuals are those with performance records together with any unobserved horses providing pedigree links between them. Summary details of all data subsets and their associated pedigrees are presented in Table 1. Note that pedigree connectedness among-traits is very high (Supplementary Table 1); sibships frequently span these distance classes and in fact many individual horses have race records across multiple classes (e.g., >30,000 individuals have both sprint and middle-distance performance records). Thus, the three data subsets do not correspond to genetically distinct management units or groups.

**Table 1:** Summary details of data subsets and associated pedigrees.

Data subset	Sample sizes			Pedigree structure							
	Performances	Horses	Races	Individuals	Sires	Dams	Max depth	Max paternal half-sibship	Max maternal half-sibship	Sires with offspring performance data	Sires with >30 offspring with performance data
Full	692,534	76,960	65,485	106,447	4169	33,859	9	1092	16	2466	542
Sprint races	317,536	53,305	29,128	78,402	3377	27,295	9	680	16	1871	448
Middle-distance races	263,067	51,610	25,235	78,177	3759	2,7627	9	1005	12	2179	427
Long-distance races	37,843	10,549	3772	22,434	2365	9833	9	491	9	1356	82

For each performance, we recorded: race identity (RaceID), finishing time, timing method (hand-timed or automatic), race distance (measured in yards; 1 furlong = 220 yards), racecourse, official ‘going’ (a measure of ground softness), the number of runners in the race (no.runners), the name, age and sex of horse, and the year of race. ‘Going’ was converted from its official (categorical) description to a numerical scale using British Horseracing Authority conversion tables (www.britishhorseracing.com/wp-content/uploads/2014/03/Going-Stick-Average-Readings.pdf). For analysis we converted the finish time (in seconds) of each individual horse to its (average) speed (yards.s^−1^) by dividing by the known race distance. Strictly this transformation assumes all horses in a race run the same (known) distance (i.e., all horses take an identical running line around the course) which will not be exactly true.

### Estimation of quantitative genetic parameters

Quantitative genetic parameters were estimated using pedigree-based animal models (Wilson et al. 2010) fitted by both restricted maximum likelihood (REML) and Markov chain Monte Carlo (MCMC) methods. For reasons we explain below, models with two different fixed effect structures were used. We refer to these as Models A and B.

### Model A structure and analyses

First, using the distance specific data subsets and their associated trimmed pedigrees we fitted three univariate animal models using ASReml (v3). These models (structure A) included a fixed factor of raceID, as well as factors of horse age and sex. Despite being continuous, we elected to fit age as a factor to condition on it without assuming any particular functional form of the average age-speed relationship. This replicates the fixed effect structure previously used to estimate genetic parameters in other racehorse populations (e.g., see Corrêa and da Mota 2007; Faria et al. 2019). In all three data subsets, fewer than 100 horses ran in age categories 12–15. Therefore, we elected to collapse later ages categories leaving age as a 10 level factor corresponding to 2–11+ years. In addition to the *additive genetic merit* (i.e., breeding value), we included random effects of *permanent environment* (i.e., non-genetic horse identity effects) and *trainer* identity. This allowed us to partition variance conditional on fixed effects into additive genetic (V_A_), permanent environment (V_PE_), trainer (V_T_) and residual (V_R_) variances. We make the standard assumption that random effects are normally distributed with means of zero and variances to be estimated, and that residuals are uncorrelated across observations. Hereafter, we will refer to this as Model A.

After fitting Model A to each distance-category data subset we estimated the heritability (h^2^) as the ratio of V_A_ to phenotypic variance conditional on fixed effects (V_P_, calculated as the sum of estimated variance components). We also tested the significance of V_A_ by likelihood ratio test (LRT) comparison to a reduced model in which the additive genetic merit was not included. We assume twice the difference in log-likelihoods is distributed as a 50:50 mix of χ^2^_1_ and χ^2^_0_ (Self and Liang 1987) which we denote χ^2^_0,1_. We then determined point estimates of the genetic contributions to improvement in speed under Model A. To do this we calculated the mean predicted breeding value (PBV) by year of birth for those horses in each dataset born between 1995 and 2012 and racing between 1997 and 2014. Mean PBV was then regressed on time (i.e., year of birth) to estimate the rate of genetic change (β_G_). We did this using PBVs for *sprint*, *middle-distance*, and *long-distance* speed traits separately.

### Model B structure and analyses

Problems were encountered attempting to fit a trivariate formulation of Model A to estimate genetic correlations of speed across distance categories. Moreover, problematically long run times were encountered when attempting to refit even the univariate model A structures in a Bayesian framework using MCMCglmm (Hadfield et al. 2010). The Bayesian analyses were required because, in contrast to REML, they permit uncertainty in PBV to be properly accounted for. It has previously been demonstrated that failure to account for uncertainly in PBVs generated under REML can lead to extreme anticonservatism with respect to statistical inference on genetic trends (though there is no necessary expectation of bias in effect size; Hadfield et al. 2010).

The computational problems encountered arose in large part from the high number of parameters entailed in fitting RaceID as a factor. Therefore we formulated a second model structure in which this effect was replaced with a set of race-level covariates previously identified as important drivers of among-race differences in average speed in our earlier work (see Sharman and Wilson 2015 for full details and justification). These were: year as a factor to ‘detrend’ the phenotype to protect against the possibility of an environmentally driven linear trend causing apparent change in mean PBVs over time (Postma 2006; Hadfield et al. 2010); fixed factors of timing method (hand-timed or automatic) and racecourse (36 level factor); quadratic functions of distance, no.runners and going; first order interactions of distance with year (as a continuous covariate), going and no.runners; and the interaction of distance^2^ with year (continuous). Horse-level effects of age and sex were included as described above for Model A and the random effect structure was unchanged.

We first fitted a univariate formulation of Model B to each distance category data subset in ASReml and verified that point estimates of temporal trends in breeding values were similar to those from Model A (see results). We were then able to successfully fit a trivariate REML model of speed in the three distance categories. We then refitted the three univariate models using MCMCglmm. For each distance category, a Markov chain of 2,000,000 iterations with a burn in of 800,000 and a thinning interval of 1200 (resulting in a posterior of 1000 samples) was used. Inverse Wishart priors with nu = 0.002 were specified for all variance components. Convergence was assessed by visual inspection of model posteriors, and by application of Heidelberg stationarity tests (*P*-values exceeded 0.05 for all variance components in all three models). We also checked autocorrelation between saved sample was low (<0.1 in all cases), and comparison of model parameter estimates to those from the corresponding REML models (very similar in all cases) provided further *post hoc* validation.

After fitting each model in MCMCglmm, we generated a posterior of the regression (β_G_) of mean genetic merit on birth year and extracted the posterior mode as our point estimate of change. We determined the posterior where β_G_ > 0 and conclude a ‘significant’ genetic contribution to phenotypic improvement if this proportion is >95%. However, noting that temporal changes in mean breeding value are expected under neutrality, we then applied a more stringent test to determine whether the estimated rate of genetic change was greater than could be explained by drift alone (following Hadfield et al. 2010). Briefly, this test uses the posterior of V_A_ to repeatedly simulate breeding values down the actual pedigree structure under an assumed absence of selection. For each of *n* = 1000 replicated simulations we then estimate the evolutionary change under drift alone as the regression of mean simulated breeding value on birth year. Using the posterior of β_G_, we calculate the proportion of times where β_G_ is greater than the simulated rate of change under drift. We conclude significant evidence for a selection response if this proportion >95%.

## Results

Univariate animal models fitted by REML under Model A confirmed the presence of low, albeit significant, levels of additive genetic variance underpinning speed (Table 2). For the three distance specific traits we obtained estimates of h^2^_sprint_ = 0.124 (0.006) (χ^2^_0,1_ = 1990.8, *P* < 0.001); h^2^_middle-distance_ = 0.122 (0.007) (χ^2^_1_ = 1913.4, *P* < 0.001); and h^2^_long-distance_ = 0.074 (0.012) (χ^2^_1_ = 115.04, *P* < 0.001). As expected from previous studies showing among-horse variance (Sharman and Wilson 2015) and trainer effects (Schulze-Schleppinghoff et al. 1985) on racehorse performance traits, V_PE_ and V_T_ were also nominally significant in all analyses (based on variance components being > 1.96 SE). Though not directly relevant to current aims, estimates of these variance components are shown in Supplementary Table 2, while model fixed effects (except raceID) are presented in Supplementary Table 3.

**Table 2:** Estimated heritabilities and temporal trends in mean predicted breeding value by birth year (β_G_) from Model A fitted by REML.

Distance	*h*^*2*^	*χ*^*2*^_*0,1*_	*P*	*β*_*G*_	*β*_*G*_ */ β*_*P*_
Sprint	0.124 (0.006)	1990.8	<0.001	0.009	60%
Middle	0.122 (0.007)	1913.4	<0.001	0.006	55%
Long	0.074 (0.012)	115.04	<0.001	0.001	17%

Values in parentheses denote standard errors. Likelihood ratio tests of V_A_ are also shown. For comparison β_G_ is also presented as a proportion of rates of phenotypic change (β_P_) reported previously. Units of β_G_ and β_P_ are yards.s^−1^.year^−1^.

Analyses of PBVs extracted from Model A yielded positive estimates of genetic trends for speed in all three distance categories (Table 2). In absolute terms the point estimate of the rate of speed improvement was greatest in sprint speed (β_G_ = 0.009 yards.sec^−1^.year^−1^) and lowest over long distances (β_G_ = 0.001 yards.sec^−1^.year^−1^). As a proportion of previously reported phenotypic trends (as estimated for the period of 1997–2012; Model 2, Sharman and Wilson 2015), the estimated genetic change is greatest over sprint distances (60% of total change as compared to 55% for middle-distances and just 17% for long-distance races; Table 2). The corresponding point estimates of β_G_ from Model B fitted by REML are identical to 3 decimal places for sprint and middle-distance race categories (Table 3). For long-distance races the point estimate of β_G_ is somewhat larger under Model B than under Model A but still remains lower than at sprint or middle distances. Post hoc checks reveal strong correlation of PBVs for individual horses across models within distance categories (*r* > 0.9 for sprint and middle-distances, *r* > 0.8 for long-distances).

**Table 3:** Estimated temporal trends in mean predicted breeding value by birth year (β_G_) from Model B as fitted by REML and MCMC.

Distance	Univariate REML	Univariate MCMC		
	*β*_*G*_	*β*_*G*_ *(95% CI)*	*P (β*_*G*_ > *0)*	*P (β*_*G*_ *>* *drift)*
Sprint	0.009	0.008 (0.0076, 0.0092)	1.000	1.000
Middle	0.006	0.006 (0.0050, 0.0063)	1.000	1.000
Long	0.002	0.002 (0.0008, 0.0029)	1.000	0.971

For MCMC estimates, 95% credible intervals are shown, as are the estimated probabilities that β_G_ is greater than zero, and that β_G_ is greater than can be explained by drift. Units of β_G_ are yards.s^−1^.year^−1^.

**Fig. 1:**
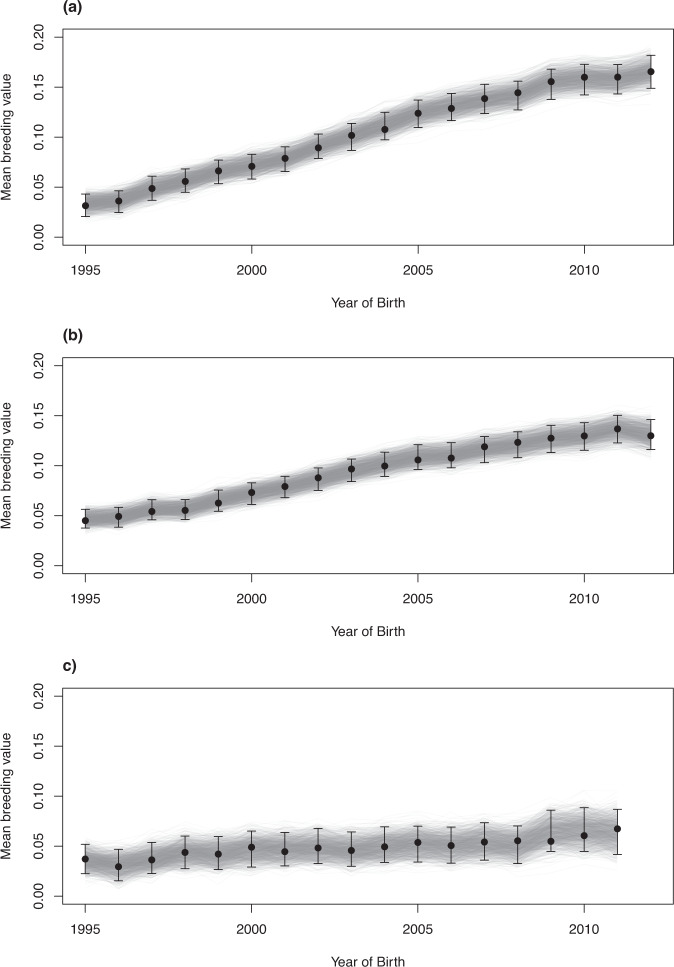
Temporal trends in predicted breeding values (PBV) for speed from Model B fitted by MCMC. Black circles indicate the posterior modes of mean PBV for speed by year of birth over **a** sprint, **b** middle and **c** long-distance races. Error bars depict 95% credible intervals. Grey lines indicate each of 1000 realisations of the temporal pattern in mean PBV.

Refitting Model B to each race distance category in MCMCglmm also yielded estimates of β_G_ that were quantitatively similar (Table 3; Fig. 1). For all data subsets, the estimated rates of genetic change are significantly greater than zero and greater than expected under neutral drift (Table 3). We therefore conclude there is evidence of genetic selection responses for speed over all distances categories.

Under Model B heritability estimates are conditional on a different fixed effect structure and are not directly comparable (but are presented for completeness in Supplementary Table 4). However, the trivariate formulation of Model B fitted with ASReml provided strong statistical support for the presence of among-distance genetic covariance structure (LRT comparison to reduced model with no genetic covariance; χ^2^_3_ = 704.4, *P* < 0.001). Estimated genetic correlations were strongly positive between speeds sprint and middle-distance speed (*r*_G_ = 0.867 (0.019)) and between middle and long-distance speed (*r*_G_ = 0.837 (0.032)). A more moderate correlation was found between sprint and long-distance speeds (*r*_G_ = 0.467 (0.006)). Assuming approximate 95% CI of *r*_G_ ± 1.96SE implies all three genetic correlation estimates are significantly greater than zero, but also significantly less than +1. More formally, LRT comparison showed the unconstrained model was significantly better than one in which we constrain r_G_ to equal +1 between each pair of traits (χ^2^_3_ = 281.34, *P* < 0.001).

## Discussion

We previously reported that thoroughbred racehorses in Great Britain are continuing to get faster (Sharman and Wilson 2015), and also showed that the rate of contemporary phenotypic improvement in GB is greatest for sprint-distance races. Here we show that the population harbours statistically significant amounts of genetic variance for speed over sprint, middle and long distances. However, our estimates of heritability are low over all distances, particularly over long-distances. We also show that the previously reported phenotypic improvement is underpinned by genetic improvement in all three distance categories. The estimated rates of improvement decrease as race distance increases. For all race distance categories, improvement rates are greater than can be reasonably be explained by drift, leading us to conclude that we are observing a selection response.

Our primary conclusion, that genetic improvement in thoroughbred racehorse speed is ongoing, contrasts qualitatively with earlier studies that argued speed was no longer increasing because the trait had reached a selection limit imposed by some form of genetic constraint (Denny 2008; Desgorces et al. 2012). Thus, while the net selection imposed by racehorse breeders is difficult to quantify (discussed further below), it is - to some extent - producing faster racehorses. Rates of genetic improvement as estimated by univariate analyses under Model A represent 60, 55 and 17% of the total phenotypic improvement over short, middle and long distances respectively. Importantly though, as noted earlier, the contemporary rates of total phenotypic improvement are themselves low. For instance, mean sprint speed is changing at an estimated rate of just +0.09% per annum relative to the observed 1997 phenotypic mean (Model 2, Sharman and Wilson 2015). This means our estimate of β_G_ = 0.009 yards.sec^−1^.year^−1^ is a genetic improvement rate of just 0.05% relative to mean sprint speed in 1997. The corresponding values for middle and long distances are 0.04 and 0.006% per annum (relative mean speeds in 1997).

Thus, while genetic improvement consistent with selection response is apparent, the estimated rates of improvement are low. As with heritability estimates, previous estimates of genetic trends for racing performance traits vary considerably. Direct comparisons are also hampered by differences in running distance, population studied, statistical methodology, modelling decisions and - perhaps especially - the performance trait modelled. Nonetheless, a study of thoroughbred racing in Japan yielded estimated rates of genetic improvement for finishing times of 1600m races in the region of 0.01–0.02% year^−1^ (Oki and Sasaki 1996) which are lower than our estimates over sprint and middle distances. Both stallions and mares were being imported to Japan at this time with the aim of improving the population (Oki and Sasaki 1996). Similar estimates were also obtained for finishing times in the Brazilian thoroughbred population (da Mota et al. 2005). This study also found genetic improvement rates were lower with increasing race distance.

Conversely, much higher rates of improvement than detected here have been reported in some previous studies. For instance, in Quarter horses, a breed which races over distances of 301–402 m, genetic improvement rates as high as 0.4% year^−1^ have been estimated (Faria et al. 2019). A previous study of thoroughbreds running predominantly in GB and Ireland generated a point estimate of the genetic improvement rate (across all distances) of about 1% year^−1^ from 1952–77 using PBV for Timeform handicap ratings (Gaffney and Cunningham 1988). This corresponds to an increase in speed of about 0.1% year^−1^ (Hill 1988). No measure of uncertainty around this trend was presented, and we note this estimate is actually greater than the total phenotypic improvement in speed as averaged across distances over historical (1850–2012) and recent (1997–2012) periods (Sharman and Wilson 2015). While noting that the authors did check the sensitivity of their trend to the assumed value of heritability, we consider it likely that both h^2^ and rate of improvement were upwardly biased by common environment effects in that case (a possibility also suggested by others: e.g., Hill 1988; Langlois 1996). Here we have tried to minimise this risk by including race level effects but also modelling any trainer influence to account for offspring of ‘better’ bred horses going to ‘better’ trainers (Schulze-Schleppinghoff et al. 1985; Schulze-Schleppinghoff et al. 1987; Hill 1988; Preisinger et al. 1989; Preisinger et al. 1990). For example, in the sprint distance REML analysis under model A, we find trainer identity explains more variance than additive genetic effects and omitting it results in estimated heritability of speed rising from 0.124 to 0.244 (full results not shown). Moreover, LRT comparison showed model A was a significantly better fit of the sprint subset when trainer was included (χ^2^_0,1_ = 8335.02, *P* < 0.001). This strongly suggests that trainer effects, if not modelled, will be a source of common environment variance that can upwardly bias V_A_ and inflate estimates of genetic change.

Our results also provide some insight into why rates of genetic improvement rates are low. In the simplest case, the univariate breeder’s equation predicts selection response as the product of heritability and linear selection differential (Lush 1937). Thus, response is limited if heritability is low and/or selection is weak. We suggest both are possible here. Conditional on fixed effects included in Model A, heritability for speed is low, particularly over long distances. The strength of selection on speed is unknown for reasons outlined above. However, by making the strong assumption that the breeder’s equation holds true, then for a trait under simple truncation selection with repeated observations per individuals, realised selection intensity *i* for each trait can be calculated as follows (Walsh and Lynch 2018):

$$i=\frac{{\beta }_{G}L}{h{\sigma }_{A}\surd (\frac{n}{1+(n-1)R})}$$

Where *β*_*G*_ is the per annum rate of improvement and *L* is the generation time (in years), ${\sigma }_{A}$ is the additive standard deviation, *h* is the square root of the heritability, *n* is the number of observations per individual, and *R* is the trait repeatability. Setting *L* to 9.2 for sprinters, 9.5 for middle-distance and 9.8 for long-distance (mean parental age at offspring birth in each data subset), *n* (mean number of records per individual) to 5.96 (sprint), 5.10 (middle-distance) and 3.59 (long-distance) and letting R = (V_A_ + V_PE_)/V_P_ then substituting in our parameter estimates from univariate analyses under Model A yields values of *i*_*sprint*_ = *1.202, i*_*middle-distance*_ = *0.905, and i*_*long-distance*_ = *0.317*. Reiterating that these are illustrative calculations made with strong assumptions, they suggest selection could be weaker than previously estimated. For example, *i*_*sprint*_ = *1.202* equates to selecting approximately 28% of the population under truncation selection. This compares to estimates of selection (across all distances) on Timeform handicap ratings of 23% (More O’Ferrall and Cunningham 1974), 32% (Field and Cunningham 1976) and 29% (Gaffney and Cunningham 1988). The lower realised selection intensities over longer races correspond to selecting >40 and >80% of the population for middle- and long-distance speed respectively.

Weak and/or inaccurate selection on speed traits may emerge cumulatively from the decision making of individual horse breeders for multiple reasons. First, speed is just one measure of performance; jockeys ride to win races, not to break records, and other phenotypic attributes contribute to a horse’s success (Langlois 1996). We have accounted for a wide range of factors in our modelling, but nuances like temperament or responsiveness to jockey controlled race tactics are unknown and unaccounted for. Nonetheless, in a racing context it is implausible that any programme of selection for increased performance (however defined) would not incorporate the aim of increasing speed. Second, there has been a general reluctance to incorporate genetic and/or genomic prediction methods (e.g., BLUP, GBLUP) in horse breeding (Hill 2016). Although low heritabilities would pose a limit to selection accuracy, such approaches still offer well documented advantages relative to selecting on phenotype. Third, since most breeders have commercial objectives, optimum sale price for resultant offspring is important. Reputation matters and ‘fashionable’ pedigrees may command higher prices regardless of actual genetic merit (Wilson and Rambaut 2008). Fourth, even given reliable information about genetic merit, cultural and economic factors limit availability of the best genes. To be allowed to race, thoroughbreds must be produced by natural matings not artificial insemination. This limits the number of offspring that can be produced each year from a given sire, while covering fees in excess of £100,000 mean leading stallions are only financially accessible to a small percentage of breeders. Fifth, selecting across multiple traits (rather than, for example, just selecting on sprint speed) is expected to reduce selection on each trait. Sixth, and rather speculatively, selection on speed may be partially countered by antagonistic selection on injury risk. It has been claimed that thoroughbreds are becoming more susceptible to injury (Drape 2008; Mitchell 2008; Gibbons 2014), perhaps as a consequence of morphology changes which have coevolved with speed (Gilbey 1903). Given additive genetic variance underpinning some thoroughbred health and conformation traits (Ibi et al. 2003; Oki et al. 2005; Oki et al. 2008; Welsh et al. 2013; Norton et al. 2016), investigation of the potential (genetic) association between injury risk and race performance would be timely. This could help to understand the evolution of speed, and may also provide tools to address ongoing welfare concerns in horseracing.

A final point emerging from our analysis is that the three distance-specific speed traits offer distinct selection targets in the sense that all pairwise genetic correlation are less than +1. We also find that the heritability of speed is lower over long-distance races, a result consistent with several other studies of finishing times (Oki et al. 1995; da Mota et al. 2005; Ekiz and Kocak 2007; Bakhtiari and Kashan 2009; Velie et al. 2015b). Thus, there is potential to improve performance across all distance categories including long distances (although there has been a commercial trend over recent decades to focus on shorter distances). The estimated correlation between sprint and long-distance performance is notably lower (*r*_G_=0.47), a result that mirrors a recent finding in Brazilian thoroughbreds (da Mota 2006). Biologically this is unsurprising; many studies have highlighted the divergence of physiological and biomechanical trait optima across running distances in human athletes (Thompson 2017). Indeed, recent work on myostatin encoding gene (MSTN) in thoroughbred racehorses has shown associations between genotype and optimal running distance that will contribute to genome-wide departures from *r*_G_ = 1 across distances (Hill et al. 2010).

In summary, we show here that speed in thoroughbred horses is heritable across categories of race distance. We also show that genetic improvement attributable to selection is contributing to previously demonstrated weak - but non-zero - rates of phenotypic improvement. However, our analyses also show that selection responses are of a limited magnitude, particularly for long-distance race performance. Low heritabilities and among-distance genetic correlation structure contribute to this pattern but weaker selection than previously assumed also seems possible. Accuracy of selection may be low across all distance categories, particularly given that modern genetic tools are rarely applied in thoroughbred breeding. This obviously contrasts with most livestock species in which much more rapid selection responses are regularly achieved.

## Supplementary information

Supplementary material

## Data Availability

The data that support the findings of this study were used under license from TBGenerations Limited, and so are not publicly available. The authors can be contacted about data access.
