# Supplementary material for: Genetic improvement of speed across distance categories in thoroughbred racehorses in Great Britain
Source: Heredity (Edinb). 2023 May 27;131(1):79–85. doi: 10.1038/s41437-023-00623-8 (PMC10313675; doi:10.1038/s41437-023-00623-8)
Supplement: Supplementary file 1 — Supplementary material [file 41437_2023_623_MOESM1_ESM.pdf]

Supplementary material

Genetic improvement of speed across distance categories in thoroughbred racehorses in Great Britain

Patrick Sharman and Alastair J. Wilson

Supplementary Table 1. Summary details of horses running in multiple distance classes, and sires and dams with offspring running in multiple distance classes.

Distance Classes	Horses	Sires with offspring spanning classes	Dams with offspring spanning classes
Sprint & Middle-distance	30,162	1,685	19,728
Sprint & Long-distance	3,986	1,095	5,769
Middle-distance & Long-distance	9,396	1,289	7,689
Sprint & Middle-distance & Long distance	3,869	1,093	5,702

Supplementary Table 2. Estimates of variance components from univariate models fitted to each dataset under Model A by ASReml. Likelihood ratio test of additive variance are described in the main manuscript while other random effects were not subject to formal statistical inference. Nonetheless, all components presented can be assumed nominally significant at P<0.05 since Z >>1.96.

**Dataset**	**Source of Variance**	**Variance (SE)**	**Z**
Sprint (5-7 furlongs)	Trainer	0.019 (0.0012)	16.5
	Additive genetic	0.017 (0.0009)	19.3
	Permanent environment	0.029 (0.0007)	41.8
	Residual	0.072 (0.0002)	341
Middle distance (8-12 furlongs)	Trainer	0.020 (0.0011)	17.8
	Additive genetic	0.015 (0.0008)	18.4
	Permanent environment	0.024 (0.0006)	37.4
	Residual	0.063 (0.0002)	302
Long distance (14-20 furlongs)	Trainer	0.010 (0.0009)	11.7
	Additive genetic	0.006 (0.0010)	5.90
	Permanent environment	0.015 (0.0010)	14.7
	Residual	0.050 (0.0005)	107

Supplementary Table 3. Estimates of fixed effects from univariate formulations of Model A fitted to each dataset using REML. Effects are considered nominally significant at P<0.05 for |Z|>1.96.

**Dataset**	**Effect**	**Performances (horses) / Effect**	**Estimate in yards.sec^{-1}.year^{-1} (3d.p) ± SE (3d.p)**	**Z**
5-7 furlongs	*sex*			
	Male	196,167 (28,832)	0 ± 0	
	Female	121,369 (24,473)	-0.049 ± 0.003	-16.3
	age (years)			
	2	120,022 (40,594)	-0.007 ± 0.04	-0.175
	3	79,786 (24,656)	0 ± 0	
	4	44,392 (10,866)	0.048 ± 0.003	16.0
	5	28,167 (5,861)	0.077 ± 0.003	25.7
	6	18,657 (3,587)	0.064 ± 0.003	21.3
	7	12,038 (2,247)	0.046 ± 0.004	11.5
	8	7,365 (1,386)	0.023 ± 0.005	4.60
	9	3,993 (743)	0.001 ± 0.006	0.17
	10	1,899 (361)	-0.040 ± 0.008	-5.00
	11+	1,217 (177)	-0.079 ± 0.010	-7.90
	mu		0 ± 0	
8-12 furlongs	*sex*			
	Male	180,143 (30,795)	0 ± 0	
	Female	82,924 (20,815)	-0.048 ± 0.002	-24.0
	age (years)			
	2	19,020 (13,300)	-0.379 ± 0.268	-1.41
	3	117,167 (38,256)	0 ± 0	
	4	55,770 (17,195)	0.089 ± 0.002	44.5
	5	30,025 (8,777)	0.038 ± 0.003	12.7
	6	17,828 (5,130)	0.037 ± 0.003	12.3
	7	10,932 (3,074)	0.021 ± 0.004	5.25
	8	6,360 (1,831)	-0.004 ± 0.005	-0.80
	9	3,325 (953)	-0.019 ± 0.006	-3.17
	10	1,600 (461)	-0.040 ± 0.008	-4.83
	11+	1,040 (227)	-0.073 ± 0.011	-6.64
	mu		0 ± 0	
14-20 furlongs	*sex*			
	Male	29,476 (7,464)	0 ± 0	
	Female	8,369 (2,985)	-0.025 ± 0.005	5.00
	age (years)			
	3	8,692 (4,651)	0 ± 0	
	4	9,820 (4,191)	-0.050 ± 0.006	-8.33
	5	6,573 (2,744)	-0.036 ± 0.007	-5.14
	6	4,940 (1,957)	-0.043 ± 0.007	-6.14
	7	3,379 (1,355)	-0.068 ± 0.008	-8.50
	8	2,195 (865)	-0.075 ± 0.009	-8.33
	9	1,205 (478)	-0.091 ± 0.010	-9.10
	10	623 (255)	-0.108 ± 0.013	-8.31
	11+	416 (135)	-0.170 ± 0.017	-10.0
	mu		0 ± 0	

Supplementary Table 4. Estimated heritabilities for speed traits from univariate and trivariate animal models fitted by REML and univariate models fitted by MCMC under Model B. Values in parentheses denote standard errors (REML) or 95% credible intervals (MCMC). Likelihood ratio tests of V_A are also shown for the univariate models fitted by REML. Note that heritabilities under Model B are lower than those reported under Model A in the main manuscript. This primarily reflects the fact that h^2 estimates are conditional on fixed effects that differ between the two model formulations (with inclusion of raceID in Model A absorbing more of the observed variance).

	Univariate REML			Trivariate REML	Univariate MCMC
Distance	h^2 (SE)	$\chi^2_{0,1}$	*P*	h^2 (SE)	h^2 (95% CI)
Sprint	0.070 (0.004)	1357.2	<0.001	0.075 (0.004)	0.068 (0.062, 0.079)
Middle	0.059 (0.004)	1051.5	<0.001	0.065 (0.004)	0.062 (0.052, 0.069)
Long	0.061 (0.010)	140.1	<0.001	0.064 (0.007)	0.062 (0.043, 0.084)
All	0.060 (0.003)	2405.0	<0.001		
